# Open‐Source Software Analysis Tool to Investigate Space Plasma Turbulence and Nonlinear DYNamics (ODYN)

**DOI:** 10.1029/2019EA001004

**Published:** 2020-04-22

**Authors:** E. Teodorescu, M.M. Echim

**Affiliations:** ^1^ Institute of Space Science (ISS) Măgurele Romania; ^2^ The Royal Belgian Institute for Space Aeronomy (BIRA‐IASB) Brussels Belgium

**Keywords:** open‐source software data analysis tool based on Python, portfolio of methods to analyze turbulence and nonlinear dynamics, the software includes visualisation tools, the software can ingest and process large collections of spacecraft data, user‐friendly parametrisation of analysis

## Abstract

We have designed and built a versatile modularized software library—ODYN—that wraps a comprehensive set of advanced data analysis methods meant to facilitate the study of turbulence, nonlinear dynamics, and complexity in space plasmas. The Python programming language is used for the algorithmic implementation of models and methods devised to understand fundamental phenomena of space plasma physics like elements of spectral analysis, probability distribution functions and their moments, multifractal analysis, or information theory. ODYN is an open‐source software analysis tool and freely available to any user interested in turbulence and nonlinear dynamics analysis and provides a tool to perform automatic analysis on large collections of space measurements, in situ or simulations, a feature that distinguishes ODYN from other similar software. A user‐friendly configurator is provided, which allows customization of key parameters of the analysis methods, most useful for nonprogrammers.

## Key Points

- We describe an open‐source software data analysis tool based on Python
- The software includes a large portfolio of methods to analyze turbulence and nonlinear dynamics and visualize the results
- The software tool is adapted to ingest and process large collections of spacecraft data as well as results of numerical simulations

## 1 Introduction

Most often, solar system plasmas are found in a turbulent state, like for instance the solar wind believed to be a universal laboratory to study turbulence (Alexandrova et al., 2013; Bruno & Carbone, 2005; Goldstein et al., 1995; Tu & Marsch, 1995). Models of isotropic incompressible turbulence (Iroshnikov, 1964; Kolmogorov, 1941; Kraichnan, 1965) partly describe properties of solar wind turbulence (see e.g., Ng et al., 2011 or Bruno & Carbone, 2005 for a review) although the solar wind is a magnetized nonisotropic plasma, which is not ideal (i.e., dissipation less) nor incompressible (Barnes*,* 1979; Hnat et al., 2005; Howes et al., 2011; Marsch & Tu, 1990; Matthaeus et al.*,* 1991). Intermittency is seen as a fundamental characteristic of variability associated with emergence of discontinuities, coherent structures leading to dissipation, heating, transport and acceleration of charged particles, or (quasi‐) inverse cascade of energy (Matthaeus et al., 2015). Significant efforts are devoted to understand the emergence of intermittency in astrophysical plasmas (Bruno et al., 2001, 2003; Burlaga, 1991; Chang et al., 2010; Consolini et al., 2005; Echim et al., 2007; Hnat et al., 2002; Marsch & Tu, 1994; Pagel & Balogh, 2001; Sorriso‐Valvo et al., 2001; Vörös et al., 2003; Wawrzaszek et al., 2015; Weygand et al., 2005; Yordanova et al., 2005). These works investigate turbulence and intermittency with a large and complex portfolio of data analysis methods, most of them based on the statistical approach or higher‐order analyses, such as probability distribution functions (PDFs), structure functions (SFs), and multifractals.

Traditionally, the presence of turbulence is indicated by a characteristic power‐law behavior of the energy spectra (i.e., power spectral density [PSD]). However, the information contained in PSD captures only partially the turbulence dynamics, especially for the cases when intermittent events are also present (Lion et al., 2016 *;* Roberts et al., 2017). Provided that the data allow for an accurate estimation of PDFs, the probabilistic description explores additional features of turbulence compared to spectral analysis. For instance, the departure of PDFs from a Gaussian shape and the increase of the flatness (the fourth‐order moment of PDFs) towards smaller scales are generally considered signatures of intermittency (Bruno et al., 2001; Marsch & Tu, 1994). The anomalous scaling of the SFs, that is, departure from self‐similarity, is the starting point for more elaborate models of turbulence based on fractals and multifractals (Benzi et al., 1993; Sreenevasan, 1991). More recently, discriminating statistical approaches have been applied to detect nonlinearity in the magnetospheric dynamics, such as mutual information (MI) or cumulant‐based cost. Such tools provide the means to relate the state of a system to its past history and thus evaluate the probability of finding the system in a particular future state (Johnson & Wing, 2005).

We envisage to narrow the gap between analysis techniques dedicated to turbulence and nonlinear dynamics, characterized by a high degree of complexity and increasingly demanding on computing resources, and the huge amount of data that is currently publicly available and is continuously collected by fleets of space missions. To accomplish this goal, we have designed and built an advanced open‐source software analysis tool—ODYN—to investigate nonlinearity, turbulence, and intermittency in the Solar System plasmas from data collected by past, current, and possibly future missions in the interplanetary space or orbiting planets of the solar system.

ODYN cumulates a rich collection of analysis methods, from lower order (PSD) to advanced (PDFs and their moments) and highly complex (multifractals and discriminating statistics). Data analysis with ODYN can be performed either on selected events or iteratively (automatic) on larger sets of measurements through an open‐source and configurable package of algorithms. These features make ODYN unique compared to similar software products dedicated to turbulence analysis, for example, Interactive Analysis Tool, developed in Matlab (http://www.storm-fp7.eu). Other Python packages designed to aid and speed up data analysis are currently under development in the field of Astronomy and Space Science, for example, AstroML—Machine Learning and Data Mining for Astronomy (Vanderplas et al., 2012) or Scikit Learn—Machine Learning in Python (Pedregosa et al., 2011) aims to create a pythonic repository of common tools, routines, and models for statistical data analysis in astronomy and astrophysics and provide a uniform and easy‐to‐use interface to freely available astronomical data sets or PySat: Python Satellite Data Analysis Toolkit that handles files and data from various sources and in different formats (Stoneback et al., 2018).

We take advantage of an extensive database dedicated to turbulence and intermittency in solar system plasmas built in the framework of the European FP7‐project STORM (http://www.storm-fp7.eu). This database includes carefully selected time series of magnetic field and plasma measurements in the solar wind at different helio‐distances and in the planetary magnetosheaths that are used to test and validate ODYN.

## 2 Prerequisites and Installation Instructions of ODYN

ODYN is compatible both with Python2 and Python3 and is available for download at http://www.spacescience.ro/projects/odyn or https://github.com/eliteodorescu/ODYN.

The Python programming language is freely available under different platforms, for example, Linux/UNIX, Windows, and MAC OS X. Complete functionality of ODYN depends on installing several key Python packages dedicated to data file reading (e.g., cdf format), array operations, or graphical representation. The complete list of necessary packages includes *numpy*, *scipy*, *matplotlib*, *jupyter*, *h5py*, *networkx*, *ffnet*, *cdf*, and *spacepy*. The simple way for setting up the environment required for the proper functioning of ODYN is to install the Anaconda package as indicated on the official website https://www.anaconda.com/download/ .

ODYN has been tested and validated on three operating systems: Ubuntu (Linux), MacOS X, and Windows. At the time of writing this Technical Report, the *Spacepy* package (Morley et al., 2011) is not fully compatible with Windows, a fact that restricts reading of *cdf* type files.

In short, the installation resides in two steps: (1) Installation of Python and *numpy*, *scipy*, *matplotlib*, *jupyter*, *h5py*, and *network* packages either manually or through Anaconda. This step is straightforward and poses no problems on either of the operating systems. This collection of libraries allows the usage of all ODYN algorithms except for the routine of reading *cdf* files, which becomes possible by following the instructions in step 2; (2) Installation of *ffnet*, *cdf*, and *Spacepy* packages requires C++ and Fortran compilers. Also, *Spacepy* supports only 32 bit architecture; thus, Python and all other libraries will also be downloaded for this architecture.

More on how to install ODYN is found in the README.txt file included in the library.

## 3 Description of the Software Library Dedicated to Space Plasma Turbulence and Nonlinear Dynamics—ODYN

### 3.1 Structure of ODYN

ODYN is a cluster of algorithms developed in Python programming language (https://www.python.org) that can be grouped largely into four main categories: (1) a collection of routines dedicated to reading and preprocessing data sets recorded by various space missions along with algorithms developed for graphical representation of these data; 2) an assemblage of pythonic implementations of both standard and advanced analysis methods dedicated to the study of turbulence and nonlinear dynamics, for example, spectral analysis, PDFs and higher‐order moments statistics, fractals and multifractals, and information theory elements; (3) the Jupyter notebook (https://jupyter.org/index.html) that facilitates the interaction with the algorithms and allows users to customize a wide array of key analysis parameters; (4) a user‐friendly configurator that facilitates an easy access to all customizable parameters and helps users without advanced programming skills to use the data analysis algorithms. Along with the code itself, test data files are provided, downloaded from the official space mission archives (ESA Cluster, ULYSSES, Venus Express).

All features of ODYN are organized in self‐explanatory folders, briefly described below.

The folder “AnalysisMethods” includes Python files (*.py*) that integrate a large portfolio of data analysis methods together with tools for data reading, data preprocessing, graphical representation, and storage. The algorithm that computes the scale‐dependent time series from a given data set, used in PDF calculations, is exemplified below.



def PDF (PDF_var, rank, scl, bin_no, compute_SF):

 DifferenceTS={}

 for s in range (len (scl)):

 ### COMPUTE TIME SERIES OF DIFFERENCES

 DifferenceTS[s]=

 np.subtract (PDF_var[scl[s]:len (PDF_var)],

 PDF_var[0:len (PDF_var)-scl[s]])

 DifferenceTS[s]=

 DifferenceTS[s][(np.abs (DifferenceTS[s])&lt;9999.)

 &amp;(np.abs (DifferenceTS[s])!=0.)]





The folder “**Data**” includes examples of data files from solar system missions like Venus Express (Svedhem et al., 2007), Ulysses (Wenzel et al., 1992), and Cluster (Escoubet et al., 1997). It also includes an example of a time series data file extracted from a particle‐in‐cell simulation that illustrates the Berstein modes in a magnetized plasma (Boyd & Sanderson, 2003). These data files allow the user to apply and test all functionalities of ODYN.

The folder “**Results**” is a repository where ODYN saves the data analysis results. This is a default output repository; the user can change it at will.

The folder “**Notebooks**” includes the Jupyter notebooks where from the user can choose the data set to be analyzed, the preprocessing technique, the type of analysis, and the parameter adjustments, data storage, etc. PDF function call is exemplified below.



“&apos;REMOVE &apos;#&apos; AND CHOOSE VARIABLE FOR WHICH PDFs WILL BE COMPUTED!!!”&apos;

#choose_PDF_var=3 ## 0,1,2, etc correspond to data(!) columns

if compute_PDF:

 PDFs,binc, Flatness,SF=AN.PDF (PDF_var,rank,scl,binno,draw_SF)





The folder “**Config**” includes a configuration structure (*Configurator*) for ODYN. The Configurator is a simple interface in the form of an ASCII file that provides the user with the means to modify a wide array of parameters specific to the analysis methods. For instance, in this folder, the user can parametrize the Welch algorithm (Welch*,* 1967) for spectral analysis and can modify the size of the Welch window, the segment length, the overlapping percent, etc., as exemplified in Table 1.

**Table 1:** Example of the Set of Adjustable Parameters That the User Can Customize to Compute, Draw, and Save the PSD

########################################################################## '''PSD ‐ COMPUTATION, PLOTTING, SAVING''' ##########################################################################		
compute_PSD	=True	# Choose whether to compute the PSD (default is True)
psd_window	='hamming'	# Choose window for Welch PSD: hamming, hanning, etc.
segment_magnitude	=512	#Choose segment magnitude for Welch PSD in number of data points
overlap_percent	=0.9	#Choose how much the segments overlap
plot_PSD	=True	# Choose whether to plot the PSDs computed for all
		# chosen data variables (default is True)
save_individual_PSD	=False	# Choose whether to save a PSD plot for each of the
		# chosen data variables (default is False)
save_allinone_PSD	=True	# Choose whether to save all PSD displayed on a single
		# plot (default is True)
##########################################################################		

Function calls or sequences of commands pertaining to ODYN are easily executed through the open‐source web application Jupyter notebook. Indeed, this is a browser‐based tool that “captures the whole computation process: developing, documenting, and executing code, as well as communicating the results” and “combines explanatory text, mathematics, computations, and their rich media output” (https://jupyter.org). The notebook designed for ODYN offers an intuitive and handy scheme for the user to call the data analysis algorithms, as detailed below.

The notebook itself is a set of cells, each cell includes sequences of commands grouped to serve a specific purpose or task (graphical representation of data, spectral analysis, etc.), which is briefly described through a cell header. Each analysis method is executed inside a dedicated notebook cell, a feature that allows users to perform one or several types of analyses by setting the variable responsible for the execution of the cell. Also, the analysis can be repeated as many times as needed with different sets of configuration parameters without starting over with data selection and preprocessing. Also, the user is guided through the algorithms with brief manual instructions meant to facilitate the interaction with the software.

### 3.2 ODYN Configurator Component

Two approaches are designed to configure or adjust parameters in ODYN: either directly in the notebook itself or through the Configurator.

The Configurator includes a large array of configurable variables like parameters defining data selection and preprocessing (e.g., *satellite* or *probe* provides the means to select data measured by different spacecraft or probes), parameters that specify how the data are represented graphically and saved (e.g., parameters *plot_data* or *save_data_fig*), etc. Some variables set by the Configurator are global, for example, the number of bins to be used in PDF computations set by parameter *binno*. Each analysis method is switched on and off through a Boolean parameter: *compute_* (i.e., compute_PSD, compute_PDF, etc.). As an example, we give below the full list of parameters that control the computation of PSD. The Configurator can be modified with the preferred text editor.

Because there is a significant number of elements available for user customization, navigation through the Configurator has been simplified by assembling elements that serve a common goal in dedicated sets. Each set is indicated with an easily identifiable visual label that is identical to the corresponding cell header in the notebook, for example, the parameters for PSD computation are grouped as presented in Table 1.

The content of the Configurator is structured on columns as exemplified above. The first column contains all the parameters that can be modified by the user (these labels cannot be changed as they will become unrecognizable by the software), the second column contains the values of the parameters (Boolean, string or numbers), and the third column contains short descriptions of the adjustable parameters. By default, ODYN will run with the parameters set in the Configurator. Yet, any parameter value modified in the notebook itself will take precedence.

## 4 Brief Description of ODYN Data Analysis Modules

ODYN includes a large portfolio of nonlinear data analysis tools, starting from the lower‐order ones (like the PSD spectrum) to higher‐order (like the multifractal spectrum). Below we review these tools that are grouped by their functionality and are assembled in a single Python file, Analysis.py, stored in the “AnalysisMethods” folder.

### 4.1 ODYN Module for Reading Spacecraft Data, Preprocessing and Graphical Representation

ODYN includes a collection of algorithms adapted to ingest data sets downloaded from official space missions' databases, such as Cluster, Venus Express, or Ulysses. ODYN includes routines adapted for reading various types of specific spacecraft data formats: *VEX_DATA_TXT()* reads “.TAB” file format specific to Venus Express mission (https://archives.esac.esa.int/psa/#!Home%20View); *ULYSSES_DATA_ASC()* is devoted to reading “.ASC” files specific to ULYSSES mission (http://ufa.esac.esa.int/ufa/); and *CLUSTER_DATA_CDF()* routine reads CLUSTER “.cdf” files (https://www.cosmos.esa.int/web/csa).

ODYN is equipped with techniques for data preprocessing and standardization that allow removal or interpolation of erroneous, missing or flagged data, and also algorithms for data trimming or selection of subsets of data. These routines can be accessed through the following function calls: (a) *CHOP_TS()*—allows the user to select a subinterval of data: The user has the possibility to input the starting and ending times, and the function selects the corresponding data that is then available for further analysis; (b) *PRINT_FLAGS()*—on‐screen printing of data flags and their position in the analyzed time series; (c) *MASK_ERR_DATA()*—flagged data are marked as “False”; this trait provides a twofold usefulness of this method: It allows for better graphical representation and prepares the data for further processing; (d) *SELF_FLAG()*—allows customization of the data flags, the user can choose the necessary flag, for example, NaN, False, 9999.9999, etc.; (e) *PSEUDOINTERP_PSD()*—applies a linear interpolation of data; this is a very useful tool for analyses that require uniform sampling of the data, such as spectral analysis. Interpolation is performed only on data gaps identified in the timeseries, the rest of the timeseries remaining unchanged.

The module dedicated to graphical representation of data is an important asset of ODYN that offers a powerful tool to quickly inspect data, easy to operate by both programmers and nonprogrammers. This module includes features such as visualization of time series and export of graphical products. It comprises the following collection of routines: *PLOT_DATA()*—for the on‐screen display of the analyzed data; *PSEUDOINTERP_PSD()*—to draw the PSD; *DRAW_PDF()*—for the graphical representation of the PDFs and Flatness; *DRAW_SF()*—graphical representation of the SFs; *DRAW_ROMA()*—this function returns two graphical outputs that include representations of intermediate and final results of the Rank‐Ordered Multifractal Analysis (ROMA); *DRAW_MI()*—for a graphical representation of the MI between two selected variables and on‐screen display of the result.

ODYN provides tools to export all on‐screen graphics; this feature can be called by assigning a “True” value to the corresponding drawing variable; for example, *save_PDF* saves the probability density functions in the default ODYN folder “Results.” Also, all on‐screen representations can be manually exported from the notebook to any user‐defined location by pressing the specific “save” icon.

### 4.2 ODYN Data Analysis Module

ODYN provides a collection of algorithms derived for a hierarchy of analysis methods dedicated to study turbulence, intermittency, complexity, and nonlinear dynamics. The user can apply these methods through specific function calls, detailed in the following subsections.

#### 4.2.1 Power Spectral Density

The *PSEUDOINTERP_PSD()* function evaluates the PSD through the Welch (1967) algorithm of averaging periodograms. A periodogram is a decomposition of the signal in trigonometric functions, see equation 1 below. The PSD gives a measure of the distribution of the energy of a variable (e.g., magnetic field or plasma bulk velocity) over ranges of frequencies. It is an extensively used tool for turbulence studies, as briefly discussed in section 1. Generally, interpolation is required for PSD evaluation (see, e.g., Munteanu et al., 2016); thus, interpolation (as described in the previous section) and PSD computation are cumulated in a single function call. Also, data stationarity is assumed for computing the PSD. Thus, a simple stationarity test algorithm is implemented in ODYN, called *Stationarity_Test()*. A Fast Fourier algorithm is applied to estimate the periodogram through a Discrete Fourier Transform of the signal. The PSD is proportional to the squared Fourier amplitude:

(1) $$S_{N}\left(\omega \right)=\frac{1}{N}{\left|\hat{X}\left(\omega \right)\right|}^{2}\;\text{where the}\;\mathrm{DFT}\;\text{is}\;\left|\hat{X}\left(\omega \right)\right|=\sum_{n=0}^{N-1}x\left(n\right)e^{-\mathrm{i\omega n}}$$

where *i* is the imaginary unit, *N* is the number of points, and *ω* is the angular frequency (*ω* = 2π/*T*, where *T* is the period). In order to reduce the error in estimating the PSD, the signal is divided into adjacent (Bartlett, 1948) or overlapping (Welch, 1967) segments, modified periodograms are computed for each segment, and the final PSD is evaluated by averaging the periodograms.

#### 4.2.2 Probability Distributions Functions (PDFs) and Their Moments, Including the SF Analysis

A probabilistic description of data is required when the data stream captures the dynamical behavior of a stochastic process. The analysis methods discussed in this subsection are based on the evaluation of a measure of variability of the physical variable X. In ODYN, such a measure is constructed from the incremental time differences of X at different time scales, τ:

(2) $$\mathrm{\delta X}\left(t,\tau \right)=X\left(t+\tau \right)-X\left(t\right).$$

The *PDF()* function achieves a computation of (i) the PDFs, (ii) their higher‐order moments, known as SFs, and (iii) the evaluation of the Flatness parameter (related to the fourth‐order moment—kurtosis).

The PDF at scale τ is estimated from the normalized histogram of the statistical ensemble of fluctuations δX obtained for that scale. The conventional method to evaluate the intermittency of the fluctuating phenomenon is to study the scaling behavior of the PDF moments, *q*, known as the SFs, defined as

(3) $${\mathrm{SF}}_{q}=〈{\left|\mathrm{\delta X}\left(\tau \right)\right|}^{q}〉=\int_{-\delta X_{\max}}^{+{\mathrm{\delta X}}_{\max}}{\left|\mathrm{\delta X}\left(\tau \right)\right|}^{q}P\left(\mathrm{\delta X},\tau \right)\mathrm{d\delta X},$$

where the integration is carried over the entire range of incremental measures (*δ*X) computed at scale *τ*. Note that the SF values for negative moment orders *q* may diverge. A quantitative measure of intermittency is the flatness parameter, *F*, computed as

(4) $$F=\frac{{\mathrm{SF}}_{4}}{{\mathrm{SF}}_{2}^{2}}.$$

We note that the binning of the histograms used for PDF estimation should be adjusted so that a meaningful representation of the analyzed data is obtained. If appropriate, the user should consider conditioning of the PDF (Kiyani et al., 2006), in dealing with extreme outliers that artificially may increase the flatness values. Also, one should be careful with the estimation of the moments of the PDF as they are affected by errors when a smaller number of samples are available from measurements (Dudok de Wit & Krasnosel'Skikh, 1996). Note also that ODYN functions take into account explicitly the presence of data gaps such that they have no incidence on the estimation of the PDF. All data gaps (flagged data or gaps in the time stamps) are identified either by checking if any data flags are present or whether the time steps exceed the nominal data resolution, in which case, a flag is added (the flag label is chosen by the user, e.g., NaN, False, numeric value, default being 9999.9999). Thus, the flagged data are excluded from further computations on the processed time series.

#### 4.2.3 Partition Function Multifractal Analysis

The multifractal approach allows for an intuitive understanding of multiplicative processes and of the intermittent distributions of various characteristics of turbulence (Grassberger & Procaccia*,* 1983; Halsey et al., 1986; Hentschel & Procaccia, 1983; Mandelbrot*,* 1989; Ott*,* 1993; Wawrzaszek & Macek, 2010). As an extension to (mono)fractals, multifractals can be seen as geometrical/mathematical objects characterized by different self‐similarities at different scales. Their relevance for multiplicative multiscale processes like the turbulent transfer of energy is evident. The multifractals can be described by an infinite number of generalized dimensions, *D*_*q*_, as depicted in (Halsey et al., 1986) and by the multifractal spectrum *f*(*α*) (Ott*,* 1993). In ODYN, the multifractal spectrum *f(α)* is computed by an algorithm based on the partition function approach (Halsey et al., 1986), implemented in the ODYN function *PartitionFunctions()*.

Given the incremental measure, *δX* (equation 2), the total interval *T* is divided into a number of segments *M = T/l* where *l = kτ*. Each segment with length *l* corresponds to a vortex with size *l*, and a new scale‐dependent normalized measure is computed as

(5) $$X_{i}\left(l\right)=\sum_{j=\left(i-1\right)k+1}^{\mathrm{ik}}X_{j}/X\;\text{with}\;X=\sum_{j=1}^{N}\delta X_{j}$$

Equation 5 associates the probability that a fraction of the fluctuations is transferred to a vortex *i* with dimension *l* from the turbulent cascade. Assuming that each normalized measure has a power‐law dependence on scale, the *q*th moment of the probabilities—the partition function—is constructed as (Macek & Szczepaniak, 2008)

(6) $$\chi \left(q,l\right)=\sum_{i=1}^{M}X_{i}^{q}\left(l\right)$$

The power‐law scale dependence of *χ(q,l),* described as *l* ^*τ(q)*^, allows for the determination of the power‐law exponents, *τ(q),* from linear fitting. *τ(q)* characterizes the fractal behavior of the subset of fluctuations that dominate the singular scaling at a specific moment order, *q*. The user should notice that the scale domain where a power‐law is identified must be chosen with care as it strongly affects the multifractal spectrum (see, for instance, a discussion by Wawrzaszek et al., 2015). Prior knowledge about power‐law behavior for different regimes of scales (from PSD or SFs analyses) helps the convergence of Partition Function Multifractal Analysis. More elaborate ways to determine the domain of scales where power‐law behavior is present are described in, for example, Macek and Szczepaniak (2008).

The generalized dimensions, *D*_*q*_, are related to the scaling exponents, *τ(q),* through:

(7) $$D_{q}=\frac{1}{q-1}\tau \left(q\right).$$

The multifractal spectrum, *f*(*α*), can be determined through the Legendre transform of the *τ*(*q*) curve (Chhabra & Jensen, 1989; Halsey et al., 1986):

(8) $$\alpha \left(q\right)=\frac{d}{\mathrm{dq}}\left[\tau \left(q\right)\right],$$

(9) $$f\left(\alpha \left(q\right)\right)=\mathrm{q\alpha}\left(q\right)-\tau \left(q\right).$$

Both functions, *f*(*α*) and *D*_*q*_, contain the same information about multifractality. However, the singularity multifractal spectrum is easier to interpret theoretically by comparing the experimental results with the models under study, see, for example, Wawrzaszek et al., 2015).

#### 4.2.4 Rank‐Ordered Multifractal Analysis

An alternative and complementary way to investigate the multifractal properties of a signal is the ROMA. This method attempts to collapse the PDFs computed for selected ranges of scales onto a single master curve (Chang, 2015; Chang et al., 2010; Chang & Wu, 2008). In ODYN, this approach is called by the function *ROMA()*.

ROMA requires the computation of the probability distributions—PDFs—and of the so‐called range limited SFs, see equation 12 below. Then, it applies a procedure similar to the one‐parameter rescaling technique (Chang, 1992; Chang et al., 2004). The one‐parameter rescaling parameter s achieves a collapse of PDFs such that the shape of the PDFs remains invariant at all analyzed scales and is described by the master curve P_s_(Y) satisfying the scaling relation given by Chang et al. (2004):

(10) $$P\left(\mathrm{\delta X},\tau \right)\tau^{s}=P_{s}\left(\frac{\mathrm{\delta X}}{\tau^{s}}\right),$$

where *Y* = *δX*/*τ*^*s*^ is a global scale invariant. If the scaling relation 10 is satisfied for all Ys, then the fluctuations at all scales pertain to a self‐similar or monofractal dynamical process. Most often, fluctuations in space plasmas cannot be rescaled by a single parameter, s (Chang et al., 2004).

ROMA emerges as a combination between the one parameter scaling methodology briefly described above and the multifractal approach. It assumes that for a multifractal ensemble of fluctuations, portions of PDFs may share the same (mono)fractal behavior; thus, these parts could be rescaled by a single parameter, according to equation 10. Based on these ideas, Chang and Wu (2008) realized that the statistical analysis should be performed on subsets of fluctuations that characterize various fractal behaviors within the full multifractal set. Therefore, grouping or ranking of the fluctuations should be performed on the rescaled fluctuations, Y. The fractal dimension of the moment order *q* is found by looking for the scaling behavior of the range limited SF:

(11) $${\mathrm{SF}}_{q}\left(\tau \right)\sim \tau^{\zeta_{q}},$$

where for a given *s* satisfying equation 10 and with *Y* = *δX*/*τ*^*s*^, the range limited SF can be written as

(12) $${\mathrm{SF}}_{q}=\tau^{\mathrm{qs}}\int_{0}^{Y_{\max}}Y^{q}P_{s}\left(Y\right)\mathrm{dY}\sim \tau^{\mathrm{qs}}.$$

From equations 4 and 5, it follows that *ζ*_*q*_ = *qs*. One also notes that the SF defined in 12 does not diverge for negative moment orders.

ROMA provides a highly complex but robust method to determine the scaling index, *s*, for subranges of the rescaled fluctuations, Y, resulting in a spectrum of scaling solutions, known as the ROMA spectrum, s(Y).

The PDF estimation is central also for ROMA; thus, the binning of the histograms must be chosen wisely. As before, the analyzed scales must pertain to a common scale regime where a power‐law is identified. Of utmost importance is also the binning and ranges of the rescaled fluctuations, Y. Attempting to collapse poorly populated portions of the PDFs or fluctuations that are not self‐affine will result in incorrect scaling indices.

#### 4.2.5 Elements of Information Theory: MI

ODYN also includes a module devoted to investigate nonlinear data correlations with information theory based methods. The ODYN function called *MI()* is devoted to an algorithm that evaluates the mutual MI between pairs of variables, (Cover & Thomas, 1991; Darbellay & Vajda, 1999; Li*,* 1990; Strehl & Ghosh, 2002). We take advantage of an existing Python implementation of MI score computation, from SciKit Learn package (Pedregosa et al., 2011), and extend the power of this tool by providing the possibility to compute and extract the baseline level through a randomization of the analyzed data (Johnson & Wing, 2005; Kugiumtzis, 1999; Theiler et al., 1992).

The MI between incremental measures computed for two scales, *δ*x(*τ*_1_) and *δ*y(*τ*_2_), is determined based on the entropies H (*δ*x), H (*δ*y), and H (*δ*x, *δ*y), computed from the marginal and joint probability distributions, respectively, that is, p (*δ*x), p (*δ*y), and p (*δ*x, *δ*y; Li, 1990):

$$H\left(\mathrm{\delta x}\right)=-\sum p\left(\mathrm{\delta x}\right)\log p\left(\mathrm{\delta x}\right),$$

(13) $$H\left(\mathrm{\delta y}\right)=-\sum p\left(\mathrm{\delta y}\right)\log p\left(\mathrm{\delta y}\right),$$

$$H\left(\mathrm{\delta x},\mathrm{\delta y}\right)=-\sum p\left(\mathrm{\delta x},\mathrm{\delta y}\right)\log p\left(\mathrm{\delta x},\mathrm{\delta y}\right).$$

By definition, the MI is given by

(14) $$\mathrm{MI}\left(\mathrm{\delta x},\mathrm{\delta y}\right)=H\left(\mathrm{\delta x}\right)+H\left(\mathrm{\delta y}\right)-H\left(\mathrm{\delta x},\mathrm{\delta y}\right)$$

The MI quantifies the information we can learn about a variable from the information contained in another variable. The nonlinear behavior present in the analyzed signal is determined with respect to a mean MI evaluated based on a number of realizations of the data with similar properties as the original signal (Kugiumtzis, 1999). Johnson and Wing (2005) propose a way to determine the reference level through a randomization procedure applied on the incremental time series that estimates the mean MI ( $\bar{\mathrm{MI}}$) and the associated standard deviation (*σ*_*MI*_) such that the final MI is estimated as:

(15) $$\frac{\mathrm{MI}-\bar{\mathrm{MI}}}{\sigma_{\mathrm{MI}}}$$

In ODYN, MI can be estimated either through equation 14 or (15).

### 4.3 Automatic Analysis in ODYN of Multiple Data Files

One of the distinguishable features of ODYN is the automatic analysis functionality developed to allow processing multiple data files from one or multiple spacecraft. This functionality allows the user to run any or all of the analysis methods implemented in ODYN iteratively, over multiple input data files from the same and/or different space missions. A dedicated notebook is designed for the automatization and stored in the “Notebooks” folder. Multiple data files can be uploaded to “/Data/AUTOMATIC/” folder, or they can be read remotely from any location as long as the user specifies the correct pathway in the notebook.

The results of the analyses are saved by default in the local folder “/Results/AUTOMATIC_ANALYSIS/.” In general, a single graphical product results after a given function call, which consists in the graphical representation of the analyzed time series and the computed objects: PSDs, PDFs, SFs, etc. The exceptions are the multifractal analyses that also produce graphical representations at intermediate steps throughout the computations in order to aid the user in choosing the optimal values of key adjustable parameters. The user can adapt the analysis parameters for the automatic analysis in the same way as for single‐file data analysis; once a set of parameters is configured, either through the Configurator or in the notebook, it will be applied on the entire collection of analyzed data. A good example of the power of such a functionality is the statistical study of the spectral behavior of the solar wind turbulent characteristics (Teodorescu et al., 2015). In this study, the python code was applied iteratively on 1,094 data files that produced a total number of 204 PSD spectra.

## 5 An Illustrative Example of Analysis With ODYN

As an illustrative example on the features of ODYN, we analyze the magnetic field fluctuations measured for about 6 hr in the magnetosheath by CLUSTER 3 spacecraft during an inbound pass.

**Figure 1:**
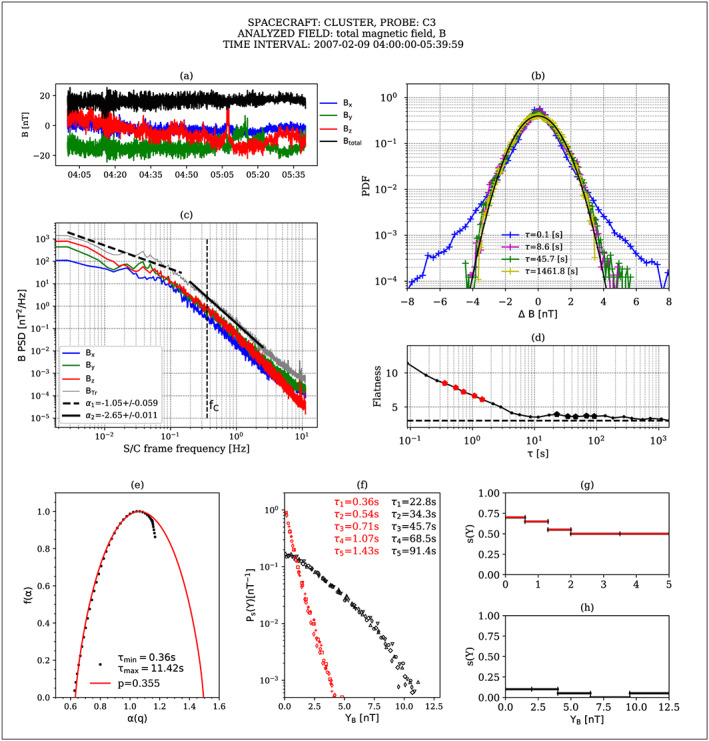
Analysis of a magnetic field fluctuations recorded in the magnetosheath, close to the **bowshock**, by the CLUSTER 3 spacecraft. (a) Magnetic field components, (b) PDFs, (c) PSD, (d) flatness, (e) multifractal spectrum f(a), (f) Ps(Y) master curve obtained by colapsing PDFs at the indicated scales with the multifractal specta shown in panels (g) and (h), different marker shapes indicate different colapsed scales, red markers correspond to small scales, black markers to large scales, evidenced also in panel (d) with larger dimond markers, (g) ROMA spectra of rescaling indices s(Y), at small scales, (h) ROMA spectra of rescaling indices s(Y), at large scales.

**Figure 2:**
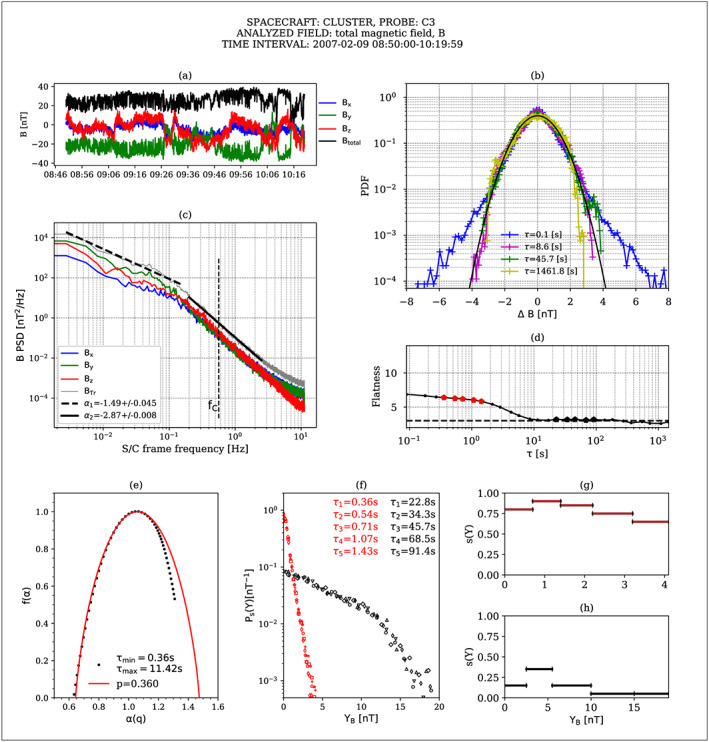
Analysis of a magnetic field fluctuations recorded in the magnetosheath, close to the **magnetopause**, by the CLUSTER 3 spacecraft. (a) Magnetic field components, (b) PDFs, (c) PSD, (d) flatness, (e) multifractal spectrum f(a), (f) Ps(Y) master curve obtained by colapsing PDFs at the indicated scales with the multifractal specta shown in panels (g) and (h), different marker shapes indicate different colapsed scales, red markers correspond to small scales, black markers to large scales, evidenced also in panel (d) with larger dimond markers, (g) ROMA spectra of rescaling indices s(Y), at small scales, (h) ROMA spectra of rescaling indices s(Y), at large scales.

We select two 1.5 hr long subintervals, one in the vicinity of the bow shock and another close to the magnetopause and deploy the suite of analysis methods implemented in ODYN. In Figures 1 and 2, we show some of the results for the *close‐to‐bow‐shock* time interval and *close‐to‐the‐magnetopause* time interval, respectively. The time series of magnetic field fluctuations are represented in panels (a) of Figures 1 and 2. The PSDs of the magnetic field components and the trace of the magnetic field spectral matrix (B_Tr_) are shown in panels (c). Linear fits of B_Tr_ for the two distinct frequency regimes are also superposed on the plots. The PDFs together with the variation of flatness with time scale, computed for the total magnetic field, B, are illustrated in panels (b) and (d), respectively. A multifractal analysis with partition functions and the ROMA is applied on the total magnetic field and the results are shown in panels (e) to (h).

The PSDs of the trace of the magnetic field spectral matrix indicate two spectral regimes/domains for both analyzed events. At frequencies below the proton gyrofrequency, the spectra are characterized by a spectral index close to f^‐1^ near the bow‐shock and steeper, f^‐1.5^, near the magnetopause, similar to the findings of Huang et al. (2017). At frequencies higher than the proton gyrofrequency, the spectra scale as f^‐8/3^ near the bow‐shock and show steeper scaling at the magnetopause. Howes et al. (2008) propose the damping of kinetic Alfvén waves through the Landau resonance as a possible cause of the steepening of the power spectrum, while Boldyrev and Perez (2012) explain an f^‐8/3^ spectrum by considering that the cascade accumulates in 2D intermittent structures. At kinetic scales, even steeper spectra have been observed and explained by solving a set of nonlinear equations (Chen & Boldyrev, 2017).

The statistical analysis of the magnetic field fluctuation adds supplementary info on the structure of turbulence. The Probability Density Functions show significant departure from Gaussian at scales smaller than 8 s (corresponding to spatial scales of the order of 1,400 km, if the Taylor hypothesis (Taylor, 1938) is satisfied). This behavior indicates the presence of intermittent fluctuations at small scales confirmed by the increase of flatness towards smaller scales as shown by panel (d). Notably, by comparing panel (d) of Figures 1 and 2, it seems the intermittent structures pertain to the same range of scales close to the bow‐shock and magnetopause, respectively. The SFs analysis (not shown) confirms the trend observed for the flatness parameter and indicates the presence of two scale domains for all moment orders, q from 1 to 5.

The multifractal analysis with partition functions results in the multifractal spectrum *f(α)*, which suggests that the analyzed fluctuations are non‐self‐similar. According to Frisch (1995), the degree of multifractality is also a measure of intermittency. Multifractal models of turbulence (e.g., Macek & Szczepaniak, 2008) can provide a fitting of *f(α)* spectrum. The width of this spectrum is considered to be a measure of multifractality and intermittency (Wawrzaszek et al., 2015). The nature of the asymmetry of *f(α)* is still under debate. An asymmetry of the singularity spectrum is sometimes thought to suggest that the turbulence is not fully developed, while other authors (Macek & Szczepaniak, 2008; Macek & Wawrzaszek, 2009; Szczepaniak et al., 2008; Wawrzaszek et al., 2015; Wawrzaszek & Macek, 2010) found that stronger intermittent pulses are associated with asymmetric multifractal singularity spectra.

We compare the multifractal spectra of the kinetic scale magnetic fluctuations measured in the magnetosheath of Earth, shown in panels (f), with a simple one parameter model, known as *p‐model* (Meneveau & Sreenivasan, 1987), and find that the best value that follows the data for positive q (left side of the singularity spectrum) is =0.355 behind the bow‐shock and 0.36 close to the magnetopause. At the subproton scales, the values of *p* might indicate a slightly wider spectrum near the bow‐shock: Δ = α_max_‐α_min_ = 0.9 as compared to the magnetopause event where *Δ* = 0.82, but the difference is very small and does not seem correlated with the measurements extracted from flatness. Nevertheless, there are clear differences between the widths of the singularity spectra between the two scale regimes analyzed for the two events, with broader spectra for the small scales, p~0.36, as compared to the spectra obtained for the larger scales, p~0.58 (not shown). Indeed, our analysis of the magnetosheath magnetic field seems to support the findings of Sorriso‐Valvo et al. (2017) for the solar wind density fluctuations that promote the idea that different measures of intermittency capture different aspects of the fluctuating field. Although these observations are in disagreement with previous results on magnetic (e.g., Kiyani et al., 2009) or density (Chen et al., 2014) fluctuations in the solar wind that indicate mono‐fractal behavior of the fluctuations at small scales, the authors identify several possible causes for the conflicting results, for example, the kinetic‐scale cascade being part of the larger magneto‐hydrodynamic (MHD)‐scale cascade, the different nature of the emerging structures in different scale regimes, or the nature of the underlying physical processes that act at various scales. Moreover, the authors stress the importance of multiple estimators for a comprehensive understanding of the analyzed phenomenon, which is also a fundamental motivation pursued for the design of the ODYN library. Our analysis further shows that the multifractality of the magnetosheath magnetic fluctuations at subion scales is confirmed also by the novel ROMA (Chang & Wu, 2008) method; more details are given in the next paragraph. The various analysis methods implemented in ODYN coupled with the possibility to analyze multiple data sets and from different spacecrafts make ODYN a powerful tool that could aid future research towards reconciling opposing results.

We select two subintervals of time scales, representative of the two scale regimes identified from the PSD and PDF analysis and perform an ROMA, Chang & Wu, 2008, for a review see Chang et al., 2010). We compute the ROMA multifractal spectrum of scaling indices, s(Y), shown in panels (g) and (h) in Figures 1 and 2. When this spectrum is applied on the PDFs of the respective scales similarly to the one‐scaling technique (Chang, 2015), one achieves a collapsing onto a single master curve P_s_(Y), shown in panels (f). The scaling index, s, similar to the Hurst exponent (Tam et al., 2010), indicates persistent or antipersistent fluctuations when it is larger or smaller than 0.5, respectively. Also, its variation with the scaled variable Y is an indication of how developed the turbulence is at the analyzed scales (Tam & Chang, 2011). Multifractal behavior of turbulent fluctuating fields (magnetic, electric) has been analyzed with ROMA for various environments in space, for example, solar wind (Chang & Wu, 2008), magnetospheric cusp (Echim et al., 2007; Lamy et al., 2008), auroral zone (Consolini & De Michelis, 2011; Tam et al., 2010), and 2 D MHD simulations (Chang & Wu, 2008).

It is clear in both analyzed cases, close to the bow‐shock or near the magnetopause, that the multifractal spectra differ significantly for the two scale regimes. At small scales, the ROMA spectra, panels (g) of Figures 1 and 2, indicate that magnetic fluctuations exhibit persistency both close to the bow shock and the magnetopause. At these scales, kinetic effects are probably responsible for the persistency of the fluctuations. Moreover, there are obvious differences between the two cases: close to the bow shock (Figure 1) s varies slower with Y, indicative of a more stable turbulent state and at large Y, it settles to a value of 0.5 characteristic to a Gaussian process. Near the magnetopause (Figure 2), s takes higher values and monotonically decreases towards larger Y but does not reach 0.5. The higher scaling indices can be correlated to the steeper scaling of the PSD, α = −2.8, which can be explained either through Landau damping (Howes et al., 2008) or may indicate the presence of intermittent structures (Boldyrev & Perez, 2012).

At large scales, panels (h) of Figures 1 and 2, the magnetic field fluctuations in Earth's magnetosheath are antipersistent, s < 0.5 (similar to solar wind, Chang et al. 2008, MHD numerical simulations, Chang & Wu, 2008) with some differences in the scaling properties of the fluctuations near the bow‐shock when compared to those near the magnetopause: while behind the bow shock a close to monofractal behavior of the fluctuations is observed, closer to the magnetopause the fluctuations appear to have a more multifractal behavior.

From a technical point of view, several features of ODYN have been used to achieve the results illustrated in Figures 1 and 2. The generic visualization module (*PLOT_DATA*) is called to select the subsets of CLUSTER magnetosheath data and plot them in panels a. The spectral module (*PSEUDOINTERP_PSD*) is called to produce the PSD spectra shown in panels c. The statistical analysis module (*PDF*) is called to compute and visualize the PDFs shown in panels b and the flatness shown in panels d. The multifractal module (*PartitionFunctions, ROMA*) is used to produce the spectra shown in panels e–h. The plots required also multiple user parameter customization, like the selected number of scales and their effective values, the binning defined for the PDF analysis, and drawing options. For the examples attached here, a user‐defined drawing canvas has been constructed, demonstrating the high versatility of the analysis software, which can easily provide publishable results and additional features have been added (e.g., PSD fitting) to the basic analyses to serve the particular analysis employed in the above example.

## 6 Summary

ODYN is a functional open source software library, written in Python, consisting in a comprehensive collection of nonlinear data analysis methods that can be applied to various spacecraft measurements. These methods proved their usefulness in studies of turbulence, complexity, and nonlinear dynamics.

The library provides several distinguishable features, like an automatic analysis of multiple data files from multiple spacecraft, detailed parameterized configuration files that allow the user a fine customization of the analysis parameters and graphical presentation of results. The library is designed to speed up and enhance the scientific output of various space missions as it offers a straight way from data to analysis results that reduces the time invested in programming itself. It also facilitates the interaction between scientists with common research interests and can be a suitable environment for sharing scientific tools, data, and double checking of results. It offers a natural framework for a collaborative development of this tool.

Automatic analysis is among the most notable assets of ODYN. Entire collections of data can be analyzed iteratively in a single run of the algorithms. The user has the possibility to choose single, several, or all of the analysis methods implemented in ODYN to be performed on the input collection of data files. As output, one obtains collections of catalogs prepared for further scientific exploitation.

Another remarkable feature of ODYN is the data visualization and preprocessing module conceived as a tool for rapid graphical representations of the timeseries/data sets. Several data formats, specific to solar system missions such as Venus Express, Ulysses, or Cluster, can be processed through ODYN while algorithms dedicated to data formats from other space missions can be easily added. Considerate effort has been invested in data preprocessing such that ODYN can guarantee output at the highest scientific quality. In this regard, several tools have been designed to manage erroneous data, data gaps, or to allow the user to self‐flag anomalous data as required.

The analysis module in ODYN spans a wide spectrum. It includes four main groups of nonlinear analysis methods: (A) spectral analysis methods (based on the PSD), (B) statistical analysis of fluctuations from time series (based on PDFs and their moments), (C) topological analysis of fluctuation with multifractals, based on ROMA and partition function formalism, and (D) elements of information theory for evaluating nonlinear correlations, for example, the MI. Any of the above analysis methods can be run independently once the set of data are prepared adequately.

A significant feature of the structure of ODYN is the Jupyter notebook designed to facilitate the interaction between the user and the modules of the library. All function calls along with the customization of analysis parameters are performed through the notebook. Additionally, analysis parameters can be adjusted and configured through the ODYN Configurator, an easily editable ASCII file, designed with the purpose of making ODYN accessible to less experienced programmers.

ODYN offers an easy access to data and to advanced analysis and visualization methods; the library is open‐access and can be deployed on several operating platforms (Linux, Mac OS, and Windows). Therefore, ODYN is meant to be exploited by experienced scientists and also as a training tool for students to get accustomed to both methodology and technical aspects of a comprehensive data analysis approach for the study of turbulence and nonlinear dynamics in space plasmas.

ODYN is ready to be integrated in online applications; there are numerous Python‐based web frameworks developed to aid the integration of Python programs as web applications, for example, Django, web2py, and Giotto. Tools like ODYN have the potential to create the context for an online database of software applications that would result in speeding up the return of scientific output of R&D activities.

ODYN fully converges to the latest philosophy of scientific publishing regarding reproducibility of research by providing the suitable environment for data, code, and figures sharing. This feature can prove its usefulness for the current international efforts devoted to publishing reproducible papers in the field of space sciences, as already accomplished in other scientific fields, for example, in February 2019, eLife announced that the first fully reproducible paper has been issued in the field of life and biomedical sciences (Blum et al., 2015; Lewis et al., 2018; Lin et al., 2012).

To our knowledge, it is for the first time when an advanced open‐source software tool provides such a comprehensive set of high‐order analysis methods dedicated to turbulence and nonlinear dynamics in space plasmas.
